# Warming modulates the photosynthetic performance of *Thalassiosira pseudonana* in response to UV radiation

**DOI:** 10.3389/fmicb.2023.1284792

**Published:** 2023-10-31

**Authors:** Zhiguang Xu, Shunda Yang, Mingze Li, Menglin Bao, Hongyan Wu

**Affiliations:** ^1^School of Life Science, Ludong University, Yantai, China; ^2^Key Laboratory of Marine Biotechnology in Universities of Shandong, Ludong University, Yantai, China

**Keywords:** ocean warming, UV radiation, photoinactivation, diatom, *Thalassiosira pseudonana*

## Abstract

Diatoms form a major component of phytoplankton. These eukaryotic organisms are responsible for approximately 40% of primary productivity in the oceans and contribute significantly to the food web. Here, the influences of ultraviolet radiation (UVR) and ocean warming on diatom photosynthesis were investigated in *Thalassiosira pseudonana.* The organism was grown at two temperatures, namely, 18°C, the present surface water temperature in summer, and 24°C, an estimate of surface temperature in the year 2,100, under conditions of high photosynthetically active radiation (P, 400–700 nm) alone or in combination with UVR (P + UVR, 295–700 nm). It was found that the maximum photochemical yield of PSII (F_v_/F_m_) in *T. pseudonana* was significantly decreased by the radiation exposure with UVR at low temperature, while the rise of temperature alleviated the inhibition induced by UVR. The analysis of PSII subunits turnover showed that high temperature alone or worked synergistically with UVR provoking fast removal of PsbA protein (K_PsbA_), and also could maintain high PsbD pool in *T. pseudonana* cells. With the facilitation of PSII repair process, less non-photochemical quenching (NPQ) occurred at high temperature when cells were exposed to P or P + UVR. In addition, irrespective of radiation treatments, high temperature stimulated the induction of SOD activity, which partly contributed to the higher PSII repair rate constant (K_rec_) as compared to K_PsbA_. Our findings suggest that the rise in temperature could benefit the photosynthetic performance of *T. pseudonana* via modulation of its PSII repair cycle and protective capacity, affecting its abundance in phytoplankton in the future warming ocean.

## 1. Introduction

It is reported that from 2011 to 2020, the average temperature of land and sea was 1.09°C higher than the average of 1850 to 1900, in which the ocean warming is 0.88°C, and the warming of the oceans will continue over time (Intergovernmental Panel on Climate Change, 2021). Scientists predict that by the close of the 21st century, surface seawater temperatures will rise by 1.18–6.48°C (Huertas et al., 2011; Gattuso et al., 2015). This will have a profound influence on ocean-dwelling organisms, as temperature significantly affects metabolic function and enzyme activity. It is predicted that the distribution and ecology of the components of phytoplankton, including dinoflagellates, diatoms and coccolithophores, would change with ocean warming (Hare et al., 2007; Hallegraeff, 2010; Cabrerizo et al., 2014; Dedman et al., 2023). Ocean warming has also been indicated to intensify stratification (Gao et al., 2019), with the upper mixed layer (UML) becoming shallower, which would influence the degree of exposure of organisms in the UML to photosynthetically active radiation (PAR) and UVR (Häder and Gao, 2015).

The release of chlorofluorocarbons (CFCs) due to human activity resulted in the depletion of ozone layer, increasing the UVR exposure of ocean surfaces (Williamson et al., 2019). Although ozone destruction has been slowed by implementation of the Montreal Protocol, with levels predicted to reach those prior to 1980 in the mid-2000s (Plummer et al., 2010), because of global warming, trace gases (Ossó et al., 2011) and other factors may elevate UV-B levels at lower latitudes (Williamson et al., 2019), retarding the restoration of the ozone layer (Neale et al., 2021). As UV irradiances can permeate the ocean surface to depths of 80 m, there will be inevitably increased exposure of phytoplankton to UVR, which could significantly affect both their richness and diversity, as well as primary productivity (Gao et al., 2019).

There has been intensive research into the independent influence of temperature and UVR on phytoplankton physiology, as has been recently reviewed (Gao et al., 2019). However, recent research has also pointed towards interactions between these factors. For example, it is reported that elevated temperature and UVR exposure significantly increased the ratio between photosynthesis and calcification in *Emiliania huxleyi* (Tong et al., 2019). Study with the diatom species *Phaeodactylum tricornutum* showed that increased temperature together with high CO_2_ level synergistically interacted to reduce UV-B-mediated inhibition, leading to increases in carbon fixation (Li et al., 2012). Wong et al. found that the damage from UVR in tropical and temperate *Chlorella* strains occurred independently of temperature although the increased temperature elevated the repair constant (Wong et al., 2015). Elevated temperature seems to ameliorate UVR-induced stress as addressed by these previous studies. Although limited studies have shown that the beneficial effects of increased temperature on phytoplankton cells in response to UVR might be related to the stimulation of excess energy dissipation (Halac et al., 2010) or enhancement of the activity of the RuBisCO enzyme (Helbling et al., 2011), the underlying mechanisms are still poorly understood.

Diatoms are single-celled photosynthetic eukaryotes that are responsible for approximately 40% of the primary productivity of the oceans (Malviya et al., 2016). They are thus key to sustain the food web and also contribute to the biological pump whereby carbon is transferred to deeper waters (Tréguer et al., 2018). Diatoms are especially plentiful at higher latitudes and in nutrient-rich coastal waters, although significant diversity of diatoms has also been seen in the open ocean (Malviya et al., 2016), indicating their ability to inhabit a variety of niches. Similar adaptability can be seen in their ability to protect themselves against potential damage caused by changes in visible light or UVR levels. In comparison with other marine phytoplankton groups, diatoms show a distinctive PSII repair cycle mechanism, characterized by the PsbD protein having a removal rate constant similar to that of PsbA (Wu et al., 2011). Diatoms can also disperse excess energy from light through rapid induction of a non-photochemical quenching (NPQ) mechanism in association with the xanthophyll cycle (Lavaud et al., 2016) together with modulation of the levels of Lhcx proteins, belonging to the antenna protein family (Taddei et al., 2018). Furthermore, diatoms have an active antioxidant system for the scavenging of reactive oxygen species (ROS) (Gao et al., 2018).

*Thalassiosira pseudonana*, as a widely distributed model diatom species, has been investigated for addressing the interaction of enhanced UVR and warming by several studies (Sobrino and Neale, 2007; Wu et al., 2020). In these studies, through measurements of Chla-fluorescence of the PSII, the influences of temperature on the rates of UVR damage and repair were specifically examined, and the results showed that ocean warming could decrease the sensitivity of *T. pseudonana* cells to UVR with an increased rate constant for repair. However, the mechanism underlying these alterations in the susceptibility of *T. pseudonana* to photoinhibition resulting from UVR exposure and increased temperature is not understood. Using our earlier studies (Yuan et al., 2018; Zang et al., 2022a,b) as a foundation, we hypothesize that high temperature may alleviate the UVR-induced photoinhibition by modulating PSII subunits turnover and photoprotection capacity. To test this hypothesis, we evaluated the photosynthetic performance of *T. pseudonana* grown at different temperatures and PAR and PAR + UVR light, investigating changes in photoinactivation, the turnover of PSII subunits, antioxidant activities, and excess energy dissipation.

## 2. Materials and methods

### 2.1. Culture of diatoms

The diatom *Thalassiosira pseudonana* (CCMP 1335) was cultured semi-continuously in f/2 medium in polystyrene flasks (Corning) with a photoperiod 12 L:12D in a growth chamber equipped with white cool tubes of fluorescent (GXZ280, Jiangnan Inc.). The cells were pre-acclimatized at either 18°C (the temperature of the local water surfaces in summer) or 24°C (the predicted water temperature in the year 2,100) with a visible light intensity of 100 μmol m^−2^ s^−1^ (20 W m^−2^). The diatoms were subcultured at least seven times, with over 20 generations, before experiments.

### 2.2. UV radiation treatment

Exponentially growing *T. pseudonana* cultures were divided and placed into two 500 mL UV-transparent quartz tubes. To allow for the determination of damage rate in PSII, lincomycin, the chloroplast protein synthesis inhibitor, was added into one of the quartz tubes to a 500 μg mL^−1^ final concentration. The quartz tubes were incubated for 10 min in the dark and were then exposed to two kinds of radiation treatments: (1) PAR + UVR (295–700 nm), full solar radiation, tubes enclosed in Ultraphan film 295 (UV Opak, Digefra, Munich, Germany) and (2) PAR only (400–700 nm), tubes enclosed in Ultraphan film 395 (UV Opak, Digefra). The settings were a PAR of 140 Wm^−2^ and UVR of 26 Wm^−2^ with adjustment of the distance of the samples from a solar simulator (Sol 1,200, Germany) with a xenon lamp (1,000 W). The tubes were incubated in a flow through water bath with controlled temperatures of 18 ± 1°C or 24 ± 1°C. Four replicates were used in each treatment. During the light exposure period, subsamples were collected at 30 min intervals for analysis of chlorophyll fluorescence, antioxidant activity, and western blotting. After 3 rounds of measurement (90 min), samples were placed under their growth-light conditions (20 W m^−2^ PAR) for 30 min before the final collection for analysis.

### 2.3. Fluorescence measurement

Samples were taken at various time points and allowed to stand for 5 min in the dark to lower photosynthesis, after which the chlorophyll fluorescence was measured with a pulse amplitude-modulated fluorometer (Water-ED PAM, Walz, Germany). The maximal PSII quantum yield was determined as the ratio of variable to maximal fluorescence, F_v_/F_m_, where F_v_ = (F_m_−F_0_), F_m_, the maximal fluorescence in darkness, measured at a 0.5 s saturating pulse of 4,000 μmol m^−2^ s^−1^; the minimal value, F_0_, was determined with modulated measuring light of less than 0.1 μmol m^−2^ s^−1^. The effective PSII quantum yield was determined using the method of Genty et al. (1989) as Φ_PSII_ = (F_m_′–F)/F_m_′, where F_m_′ is the instantaneous maximum intensity of fluorescence in an light-adapted cell measured by a saturating pulse in the presence of a weak actinic light (100 μmol m^−2^ s^−1^), and F is the steady-state fluorescence induced by weak actinic light for light-adapted cells. The quantum yields of constitutive, energy-independent non-photochemical excitation energy dissipation (Y_(NO)_) and energy-dependent, regulated non-photochemical excitation energy dissipation (Y_(NPQ)_) were calculated using the equations Y_(NO)_ = F/F_m_ and Y_(NPQ)_ = 1–Φ_PSII_-Y_(NO)_, respectively, according to Klughammer and Schreiber (2008). The sustained NPQ (NPQs) was calculated as: NPQs = (F_mt0_−F_m_)/F_m_, where F_mt0_ represents the F_m_ value measured from samples taken before the high-light treatment, with measurement of F_m_ at different time points after incubation in the dark for 5 min (Wu et al., 2012).

The rate constants for photoinactivation (K_pi_, s^−1^) and for PSII repair (K_rec_, s^−1^) were determined according to the plot of F_v_/F_m_ against the total photons present during the high-light treatment. K_pi_ was acquired from samples treated with lincomycin via plotting a single-phase exponential decay. K_rec_ was estimated from samples not treated with lincomycin. These were calculated using the Kok equation (Kok, 1956; Campbell and Tyystjärvi, 2012).

### 2.4. Protein measurement

Samples (30 mL) were harvested at different time points and vacuum-filtered using 25 mm diameter, binder-free Whatman GF/F glass fiber filters before rapid freezing in liquid nitrogen and storage at -80°C until use. Total protein was isolated from the frozen filters following Brown et al. (2008) and the concentrations were measured (Bio-Rad DC Assay). Two micrograms of protein were electrophoresed on 6–12% acrylamide gels to isolate PsbA and PsbD. The primary antibodies were anti-PsbA (1: 50,000; Agrisera, antibody AS05084) and anti-PsbD (1: 50,000; Agrisera, antibody AS06146) with an HRP-conjugated anti-rabbit secondary antibody. The removal rate of PsbA (K_PsbA_, s^−1^) was assessed as in our previous study (Gao et al., 2018).

### 2.5. Superoxide dismutase and catalase activities

Twenty milliliters of culture was filtered over 0.22 μm proe-size polycarbonate membranes (Whatman). Filters were thawed and resuspended in 600 μL of phosphate buffer (pH 7.6) containing 50 mM KH_2_PO_4_, 1 mM EDTA, 0.1% Triton X-100, and 1% (w/v) polyvinylpolypyrrolidone. The cells were ultrasonicated (2 × 30 s, at A = 30) at 4°C followed by centrifugation for 10 min (12,000 g, 4°C). Supernatants were collected into clean Eppendorf tubes and the protein quantified by the method of Bradford (1976). SOD activity was examined using a kit (Nanjing Jiancheng Biological Engineering Company, China) with 1 unit defined as the amount required to achieve 50% inhibition of nitro-blue tetrazolium reduction at 560 nm (Janknegt et al., 2008). The same sample treatment was used for measuring CAT, which was determined using a kit (Nanjing Jiancheng Biological Engineering Company) and assessed by H_2_O_2_ consumption at 240 nm (Li et al., 2015).

### 2.6. Statistical analysis

SPSS version 22.0 was used for data analysis. The effects of light exposure on PsbA, PsbD and F_v_/F_m_ was assessed by RM-ANOVA while the effects of temperature, lincomycin, and UVR were evaluated by multivariate ANOVA. Tukey’s *post hoc* test was used. Two-way ANOVAs were used to evaluate the effects of temperature and UVR on K_rec_, K_pi_ and K_PsbA_. For all tests, significance was set at *p* < 0.05.

## 3. Results

**Figure 1:**
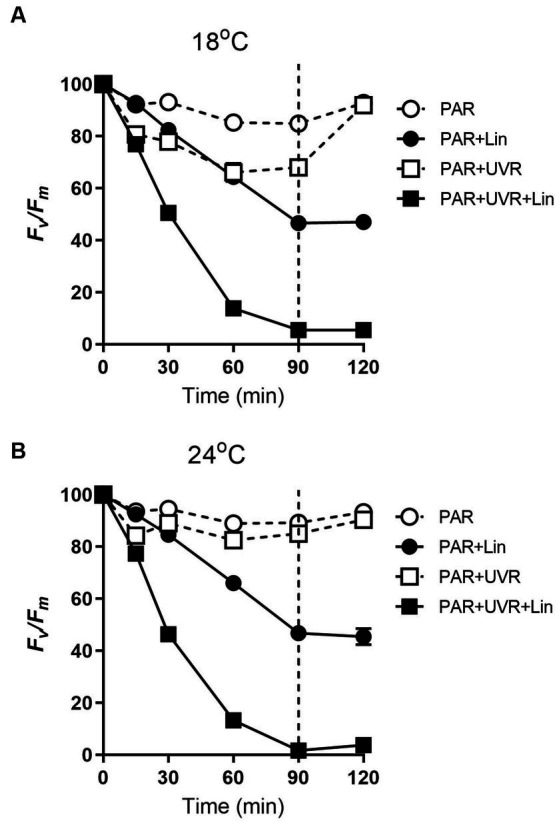
The maximum photochemical yield (F_v_/F_m_) changes in *T. pseudonana* treated without or with lincomycin (+Lin). Cells grown at 18 **(A)** or 24°C **(B)** were exposed to PAR (140 W m^−2^) or PAR + UVR (140 W m^−2^ + 26 W m^−2^) for 90 min, and then transferred to PAR (20 W m^−2^) for 30 min. The division between high light exposure and recover period was indicated by the dotted line. Data are expressed as the means±SE (*n* = 4), most error bars within symbols.

The PSII function (F_v_/F_m_) in *T*. *pseudonana* cultured at both temperatures (18°C and 24°C) decreased with exposure time under high PAR or PAR + UVR (*p* < 0.001), also under conditions of active PSII repair (Figure 1). After exposure for 90 min, the F_v_/F_m_ ratios were reduced to 85% for PAR and 68% for PAR + UVR at 18°C, while at 24°C, there was a smaller reduction to about 89% for PAR and 85% for PAR + UVR. In terms of lincomycin treatment, there was a sharper decrease in the F_v_/F_m_ value over 90 min at both temperatures (all treatments, *p* < 0.001), while in the UVR-treated cells (*p* < 0.001), the F_v_/F_m_ dropped to close to 0. Following transfer to low-light conditions, F_v_/F_m_ increased to 93% of the initial value at both temperatures irrespective of the radiation treatments in the absence of lincomycin. The cells treated with lincomycin did not recover (for all treatments, *p* < 0.001).

The rate constants for photoinactivation K_pi_ (s^−1^) and PSII repair K_rec_ (s^−1^) are given in Table 1. The rise of temperature had no effect on K_pi_ (*p* = 0.362), while UVR treatment led to a significant increase in K_pi_ to a value approximately 3.3 times that of PAR. However, an interaction between UVR and temperature was apparent for K_rec_ (*p* < 0.01), with temperature showing the dominant effect (*p* < 0.001), and the greatest K_rec_ value was observed with PAR + UVR treatment at 24°C. UVR caused much lower ratio of K_rec_ to K_pi_ at 18°C, while the ratio increased 2.9 times at 24°C.

**Table 1:** The rate constant for PSII repair (K_rec_, s^−1^) and photoinactivation (K_pi_, s^−1^) and the ratio of K_rec_ to K_pi_ for various treatments.

Temperature (°C)	Radiation treatments	K_rec_	SE	K_pi_	SE	K_rec_/K_pi_
18	PAR	0.000851^a^	0.000119	0.000122^a^	0.000006	6.96
24	PAR	0.001003^a^	0.000168	0.000129^a^	0.000008	7.77
18	PAR + UVR	0.000981^a^	0.000087	0.000415^b^	0.000023	2.36
24	PAR + UVR	0.002888^b^	0.000273	0.000424^b^	0.000024	6.81

Data are the means ± SE of four replication. Superscripts with different letters represent significant differences (*p* < 0.05) among treatments.

**Figure 2:**
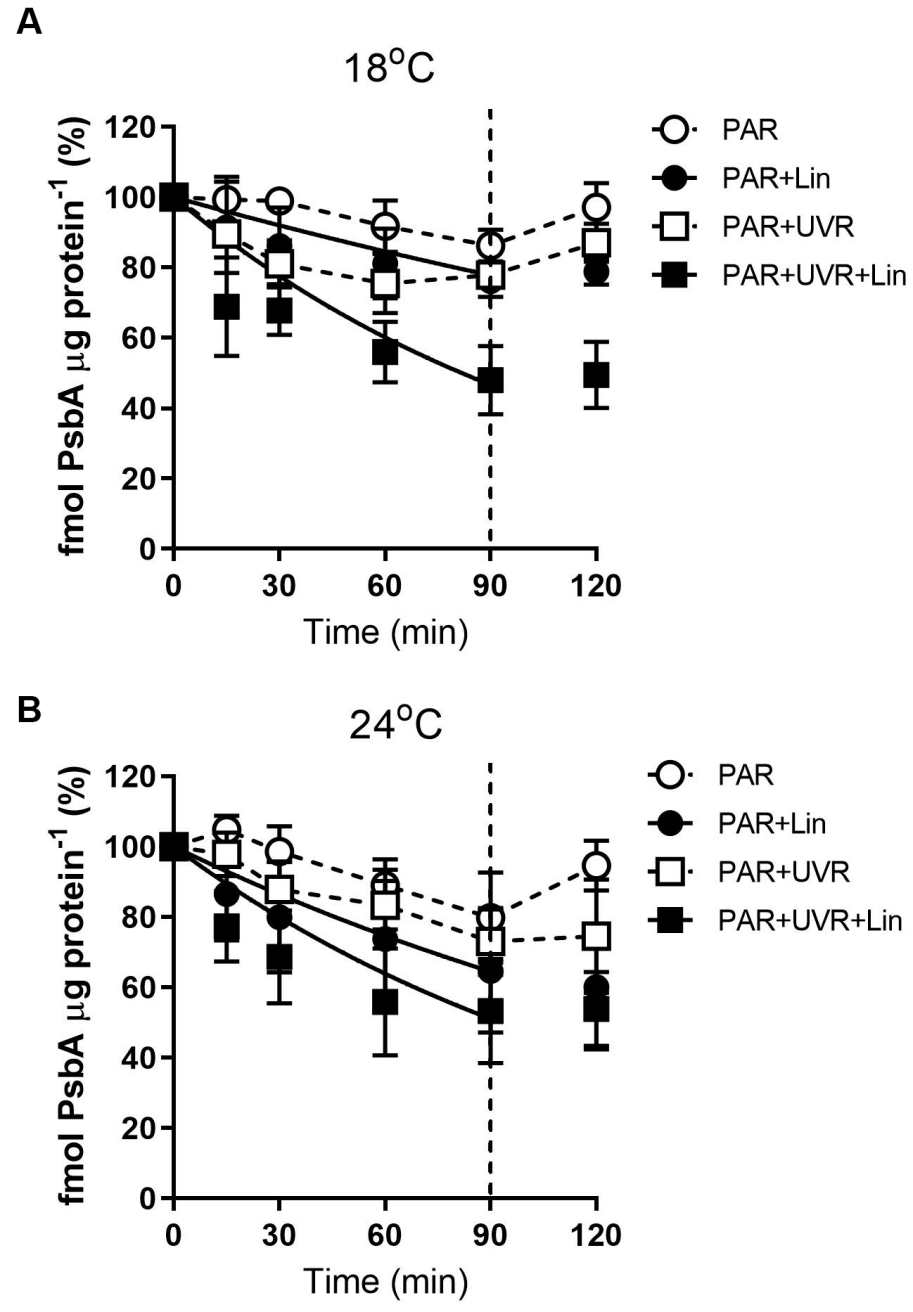
PsbA content changes in *T. pseudonana* treated without or with lincomycin (+Lin). Cells grown at 18 **(A)** or 24°C **(B)** were exposed to PAR (140 W m^−2^) or PAR + UVR (140 W m^−2^ + 26 W m^−2^) for 90 min, and then transferred to PAR (20 W m^−2^) for 30 min. The division between high light exposure and recover period was indicated by the dotted line. Data are expressed as the means±SE (*n* = 3).

The levels of PsbA were reduced after prolonged PAR and PAR + UVR treatments (Figure 2, *p* = 0.017), dropping to about 86% of time 0 under PAR after 90 min exposure for cells grown at 18°C, while a further decrease to 76% under PAR + UVR. For cells grown at 24°C, PsbA content showed a similar declination during the 90 min exposure with no significant changes observed between PAR and PAR + UVR (*p* = 1.000). In contrast, PsbA levels were markedly reduced after incubation with lincomycin (*p* < 0.001). After placing the cells under growth-light conditions, the PsbA content increased to 94–97% of the time 0 value after exposure to PAR, while there was no recovery seen in cells after lincomycin treatment.

**Figure 3:**
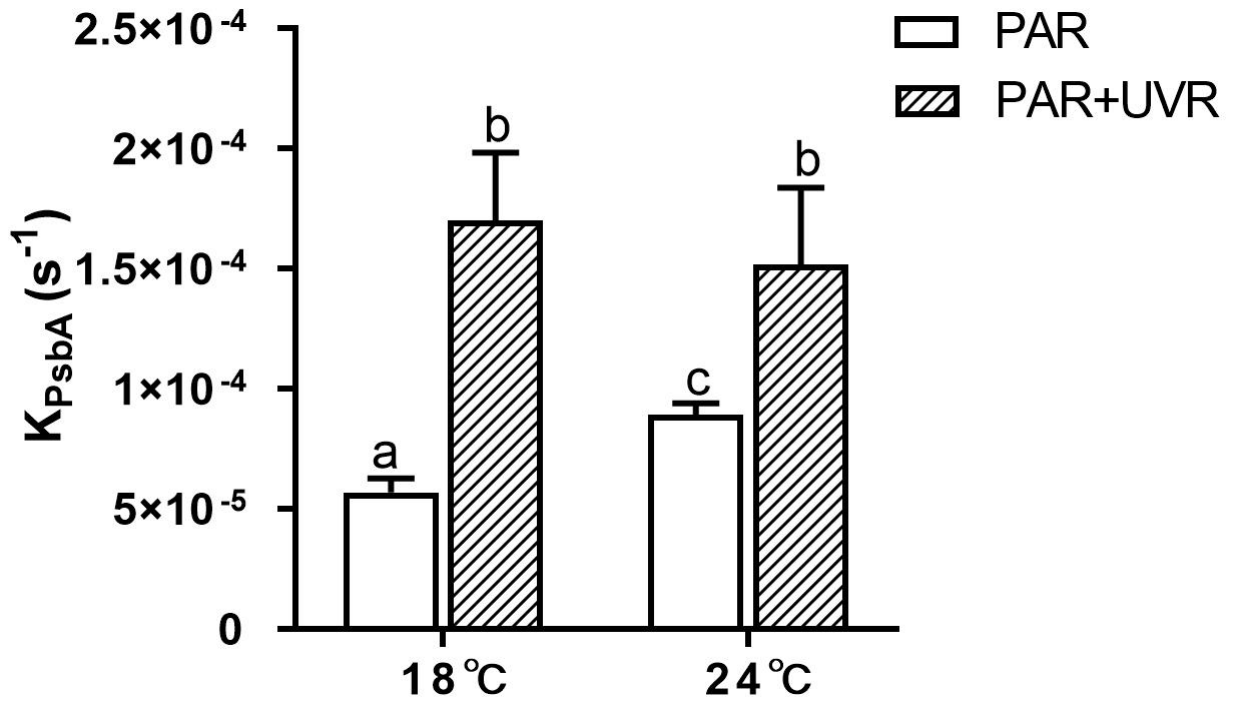
The removal rate constant of PsbA (K_PsbA_, s^−1^) in *T. pseudonana* grown at 18 or 24°C. Data are expressed as the means ± SE (*n* = 3). Different letters above error bars indicate significant differences (Tukey HSD), (*p* < 0.05) among treatments.

The rate constant for PsbA removal (K_PsbA_) is shown in Figure 3. K_PsbA_ was significantly increased by the radiation treatment in the presence of UVR (*p* < 0.001), cells cultured at 24°C showed a significant increase of K_PsbA_ as compared with that at 18°C under the exposure of PAR (*p* < 0.001). However, the combination of high temperature and UVR did not provoke further stimulation of K_PsbA_ as compared with that at 18°C.

**Figure 4:**
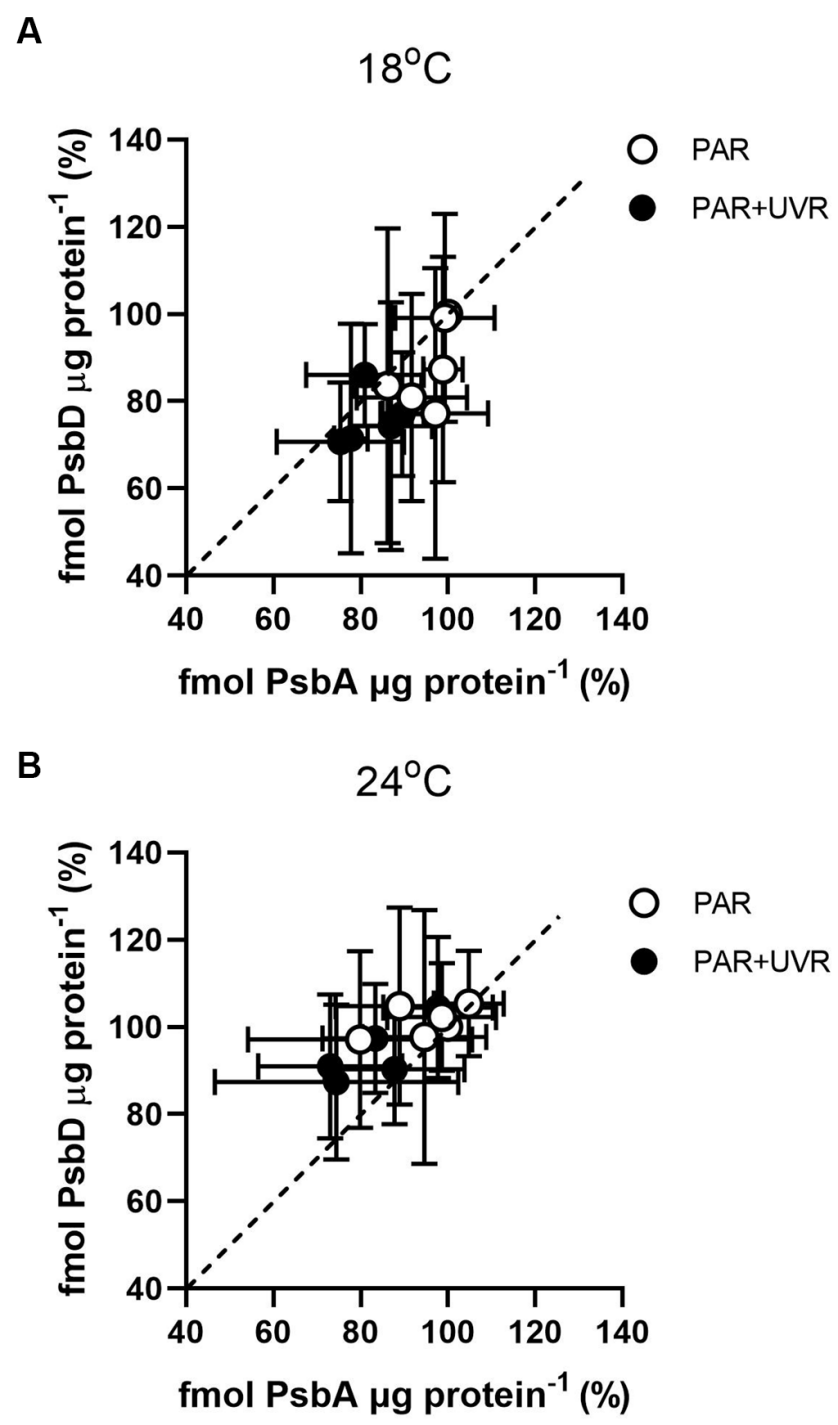
PsbD content versus PsbA content in *T. pseudonana* grown at 18 **(A)** or 24°C **(B)** over a 90 min exposure to PAR and PAR + UVR and subsequent recovery at growth light. Dashed line indicates 1:1 ratio. Data are expressed as the means ± SE (*n* = 3).

Figure 4 shows the changes of PsbA vs. PsbD content during the 90 min exposure. The turnover of PsbD was similar to that of PsbA, which was to be expected as the two protein assemble in a 1:1 ratio in the PSII complex. However, it exhibited a higher PsbD content relative to PsbA in *T. pseudonana* cells grown at 24°C, while higher PsbA content than PsbD in cells grown at 18°C.

**Figure 5:**
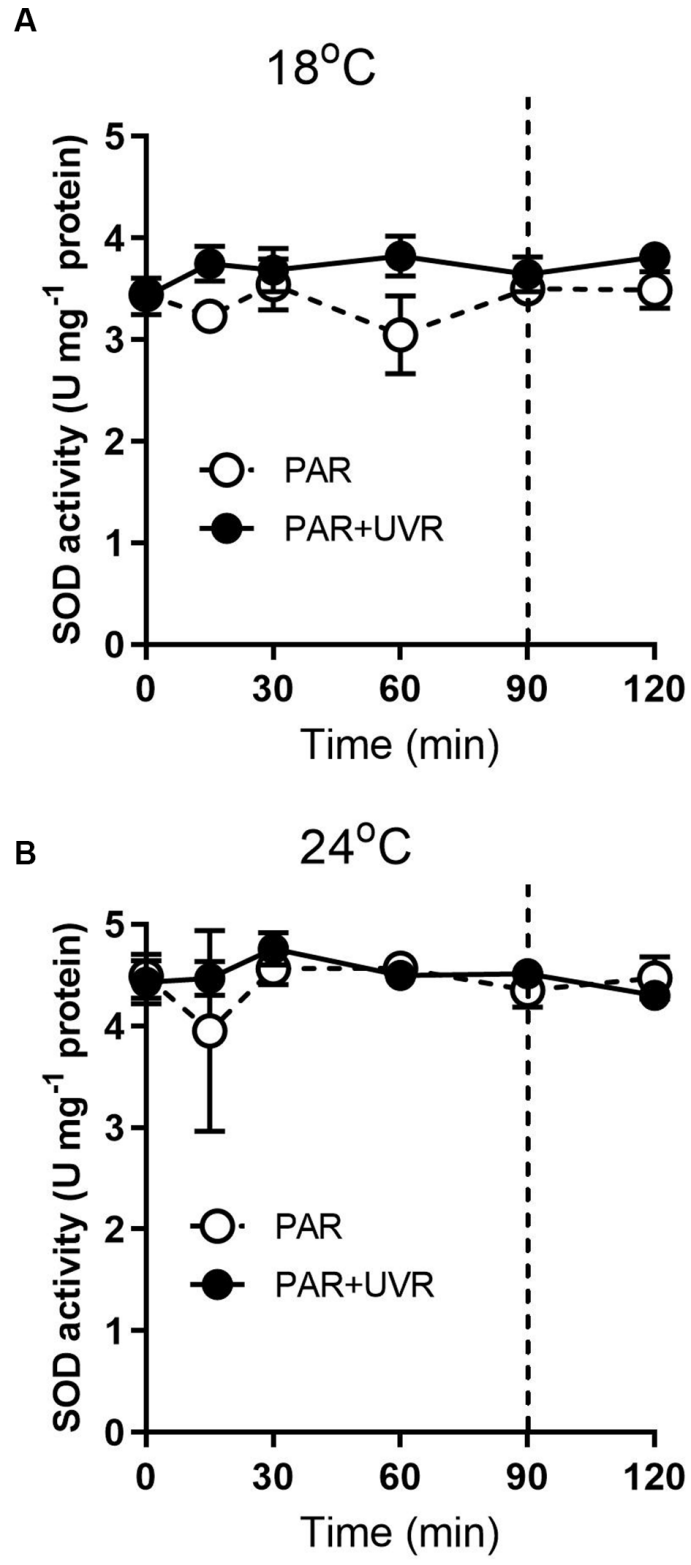
SOD activity changes in *T. pseudonana* grown at 18 **(A)** or 24°C **(B)**. Cells were exposed to PAR (140 W m^−2^) or PAR + UVR (140 W m^−2^ + 26 W m^−2^) for 90 min, and then transferred to PAR (20 W m^−2^) for 30 min. The division between high light exposure and recover period was indicated by the dotted line. Data are expressed as the means±SE (*n* = 4).

**Figure 6:**
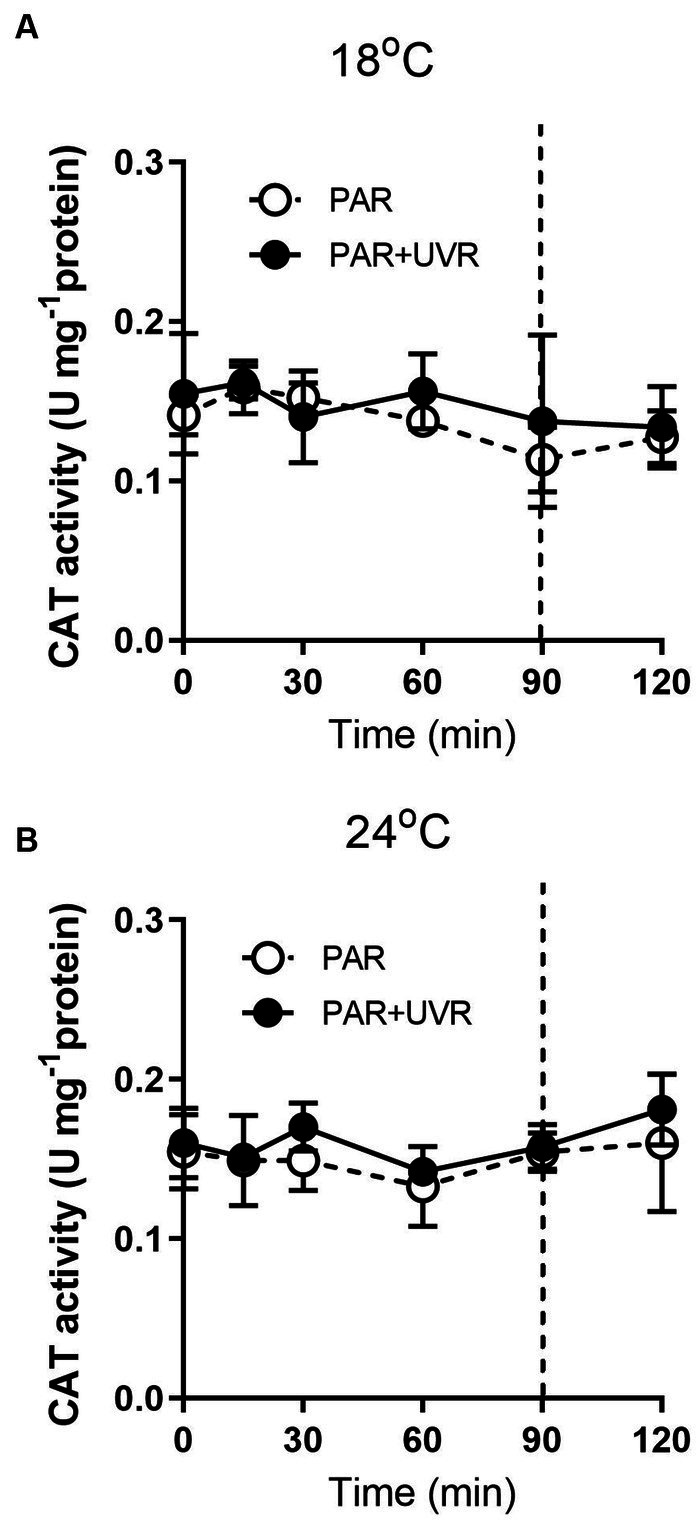
CAT activity changes in *T. pseudonana* grown at 18 **(A)** or 24°C **(B)**. Cells were exposed to PAR (140 W m^−2^) or PAR + UVR (140 W m^−2^ + 26 W m^−2^) for 90 min, and then transferred to PAR (20 W m^−2^) for 30 min. The division between high light exposure and recover period was indicated by the dotted line. Data are expressed as the means±SE (*n* = 4).

For cells grown at 18°C, UVR led to a slight increase of SOD activity than that of PAR alone (Figure 5, *p* < 0.001). It showed that the activity of SOD was higher in cells grown at 24°C than that at 18°C (*p* < 0.001), and maintained stable during the high light exposure and the subsequent recovery period with a lack of significant differences between PAR and PAR + UVR (*p* = 0.639). In contrast, there is no significant changes in CAT activity over the whole exposure period with or without UVR for cells grown at both temperatures (Figure 6, for 18°C, *p* = 0.718; for 24°C, *p* = 0.393).

**Figure 7:**
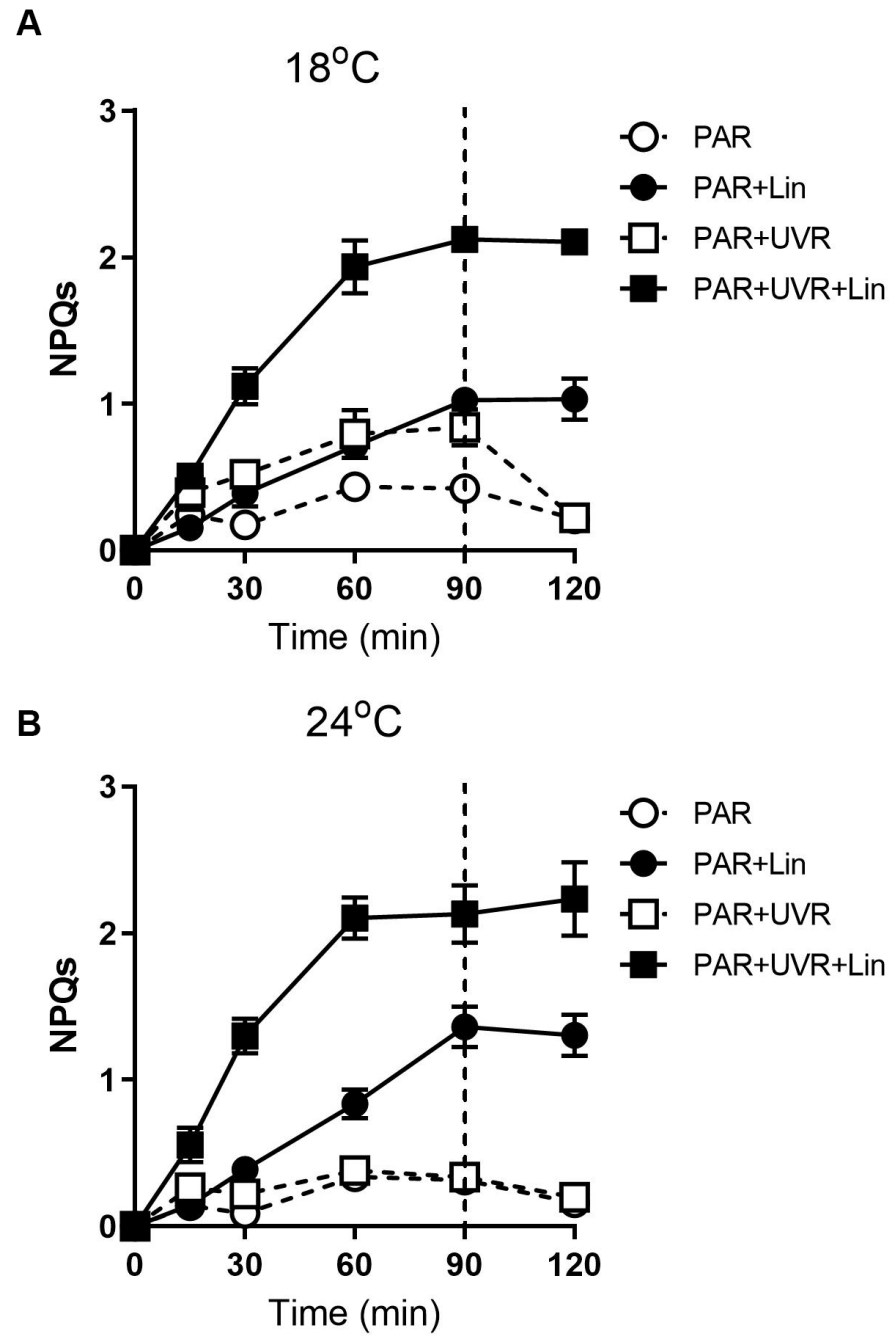
The sustained NPQ (NPQs) changes in *T. pseudonana* treated without or with lincomycin (+Lin). Cells grown at 18 **(A)** or 24°C **(B)** were exposed to PAR (140 W m^−2^) or PAR + UVR (140 W m^−2^ + 26 W m^−2^) for 90 min, and then transferred to PAR (20 W m^−2^) for 30 min. The division between high light exposure and recover period was indicated by the dotted line. Data are expressed as the means±SE (*n* = 4).

Extended exposure to light resulted in increased NPQs (Figure 7, *p* < 0.001) with a greater effect seen with UVR at 18°C (*p* < 0.001). The high temperature decreased the NPQs compared to the low temperature (*p* = 0.048), and no additional NPQs induction was promoted by UVR in cells grown at 24°C. NPQs were also induced by lincomycin treatment (*p* < 0.001), especially for cells exposed to PAR + UVR (*p* < 0.001). Following recovery, there was a reduction in the NPQs in cells that had not been treated with lincomycin, while no change was seen in lincomycin-treated cells.

**Figure 8:**
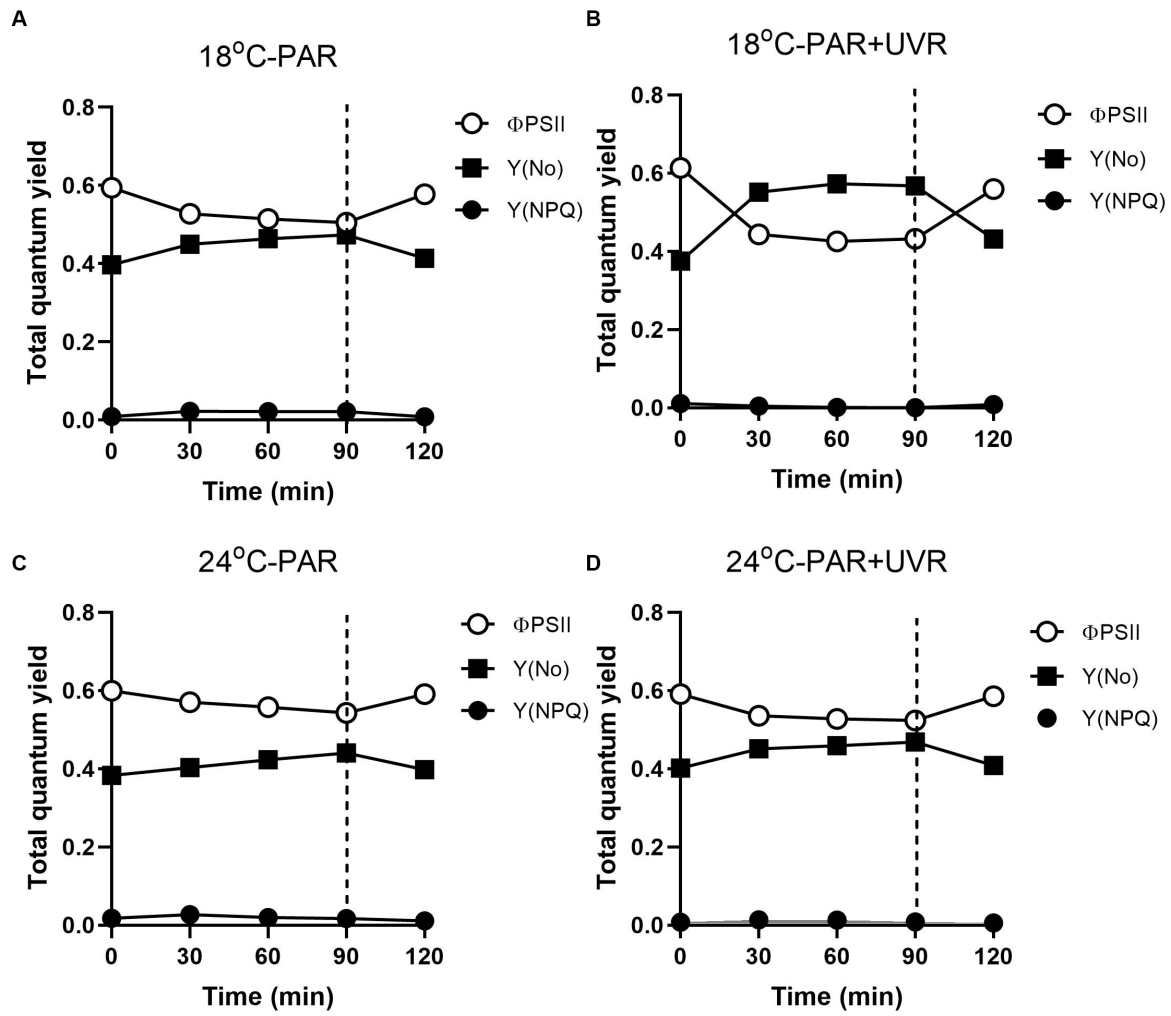
Changes of the effective PSII quantum yield (Φ_PSII_), quantum yield of regulated non-photochemical excitation energy dissipation (Y_(NPQ)_), and quantum yield of non-regulated non-photochemical energy dissipation in PSII (Y_(No)_) in *T. pseudonana*. Cells grown at 18 **(A, B)** or 24°C **(C, D)** were exposed to PAR (140 W m^−2^) or PAR + UVR (140 W m^−2^ + 26 W m^−2^) for 90 min, and then transferred to PAR (20 W m^−2^) for 30 min. The division between high light exposure and recover period was indicated by the dotted line. Data are expressed as the means±SE (*n* = 4).

Figure 8 shows the excitation energies at the two temperatures. Similar patterns of energy reallocation were seen with reduced PSII photochemistry (Φ_PSII_) following exposure to PAR, although the non-regulated dissipation (Y_(No)_) was raised. There was a reversal of these changes in the recovery period, although there was minimal allocation on regulated dissipation (Y_(NPQ)_). With PAR, compared with cells grown at 18°C, cells grown at 24°C showed more reallocation of excitation energy to Φ_PSII_ (*p* = 0.004) and less to Y_(No)_ (*p* = 0.037), although no significant alteration in Y_(NPQ)_ was seen (*p* = 0.643) by the end of the exposure. However, PAR + UVR resulted in a marked energy reallocation to Y_(No)_ with reduced Φ_PSII_ in cells at 18°C (*p* < 0.001), while at 24°C, Φ_PSII_ decreased to a less extent (*p* = 0.162) with a small increase in Y_(No)_ (*p* = 0.086) as compared with the values at time 0, in addition, the reallocation of Y_(NPQ)_ was dropped to almost zero at both temperatures.

## 4. Discussion

The effects of increased UVR and their influence on temperature-dependent physiological processes are of particular importance under conditions of global warming. Here, the F_v_/F_m_ ratio, representing the photochemical quantum yield, of *T. pseudonana* at current normal temperatures (18°C) was markedly decreased by exposure to high PAR and PAR + UVR, with further declines with UVR (Figure 1A), while the higher temperature (24°C) mitigated the reduced PSII activity under both treatments (Figure 1B). Similar results were also found in the previous studies, for example, by using the same diatom species *T. pseudonana*, Sobrino and Neale found that the high temperature of 25°C decreased the sensitivity of cells preacclimated to 20°C to UVR (Sobrino and Neale, 2007). Other diatom species, such as *Chaetoceros gracilis* and *T. weissflogii*, have been found to benefit from increased temperature (23°C), leading to reduced photoinhibition relative to samples treated at 18°C (Halac et al., 2010).

It is suggested that the alleviation of UVR-induced inhibition might be related to the facilitation of repair process by warming as indicated by the increased value of rate constant for repair, which was obtained based on the measurement of chlorophyll fluorescence (Wong et al., 2015; Wu et al., 2020). Here, alterations in PsbA contents were evaluated to assess the subunit turnover and restoration of of functional PSII complexes. It was found that the PSII function declined more rapidly than the PsbA content in cells cultured at 18°C, particularly for those exposed to UVR, suggesting the presence of photo-inactivated but otherwise intact PsbA subunits. However, on exposure to PAR and PAR + UVR, the overall PSII function increased while the PsbA level declined in cells cultured at 24°C, suggesting a modulatory response involving increased net clearance of PsbA from photo-inactivated but intact PSII complexes (Gao et al., 2018), which is also suggested by the observed increases in the PsbA removal rate constants (K_PsbA_, s^−1^) in cells cultured at 24°C. The stimulation of UVR on the clearance of PsbA, as found in our previous studies with *T. weissflogii* and *Skeletonema costatum* (Gao et al., 2018; Zang et al., 2022b), might act as a means of protection as rapid removal of damaged PsbA may facilitate the recruitment of of new PsbA. However, the combination of high temperature and UVR did not provoke further stimulation of K_PsbA_ as compared with that at low temperature, suggesting that there may be restrictions on the ability of cells to remove PsbA from photo-inactivated PSII. PsbD is thought to function as a receptor for newly synthesized PsbA subunits, thus contributing to the regulation of complex assembly (Komenda et al., 2012). We found here that cells grown at 24°C maintained higher PsbD protein content than PsbA under PAR and PAR + UVR during the exposure period. The raised levels of PsbD could thus function as a reserve derived from disassembly of PSII complexes that could be used for future combination, thus maintaining the levels of functional PSII, which may also partly account for the higher K_rec_ values seen at increased temperature, as well as the increase in F_v_/F_m_ (Figure 1B).

Apart from the PSII subunit turnover, *T. pseudonana* exposure to increased light necessitates dissipation of the surplus energy to avoid damage to PSII. In the present study, NPQs increased with exposure time, particularly for cells exposed to UVR at low temperature, suggesting the presence of protective strategies to counteract the excess energy, which might be associated with increased expression of Lhcx protein (Mou et al., 2012; Wu et al., 2012; Allorent et al., 2017). This increase was greater after treatment with lincomycin, a dependence on repair of the PSII complex to reverse the damage; thus, NPQs are important for the maintenance of PSII in the absence of repair to the complex. High temperature induced less NPQs accumulation as compared with low temperature of 18°C in the present study, correlating to the fast removal rate of PsbA and the maintenance of higher PsbD pool for cells grown at 24°C. In addition, it is reported that in *T. weissflogii* the levels and activity of RuBisCO were enhanced at high temperature when exposed to UVR (Helbling et al., 2011), and our previous study with the same diatom species *T. pseudonana* also showed that the RuBisCO to PsbA ratio was raised after exposure to high light; therefore, it could be deduced that more photons would be processed in the Calvin cycle for *T. pseudonana* cells, requiring less energy dissipation.

In terms of PSII excitation energy, UVR was found to reduce the amount of photochemical energy in PSII (Φ_PSII_) at the lower temperature (Figure 8), with greater allocation to Y_(No)_, in other words, greater passive dissipation of energy as fluorescence and heat, indicating the weak ability of cells to protect themselves against photodamage. In contrast, *T. pseudonana* allocated much less excitation energy to Y_(NPQ)_ under all treatments, particular for cells exposed to UVR, representing an energy-dependent type of NPQ associated with the xanthophyll cycle (Liefer et al., 2018). It might be related to negative effects of UVR on the enzymes involved in xanthophyll conversion as found in *Phaeodactylum tricornutum* (Mewes and Richter, 2002; Halac et al., 2009). It was reported that when the activation of xanthophyll cycle was not sufficient to quench the excess energy, cells can reduce ROS levels through the action of antioxidant enzymes (Janknegt et al., 2008). We found that the antioxidative defence system was activated, mainly through the action of SOD, UVR induced a slight increase of SOD activity at low temperature than PAR, which is able to scavenge the superoxide radical and contribute to the prevention of protein damage. Cells produced higher induction of SOD activity at high temperature irrespective of radiation treatments, showing the enhanced scavenging capacities to excess light, which might partly explain the higher K_rec_ compared to K_PsbA_. Since CAT converts the H_2_O_2_ product of SOD to H_2_O, the activity of the two enzymes would be expected to increase in correspondence, but this was not observed here. The similar uncoupling phenomena was also reported in some marine intertidal diatom species (Bettina et al., 2014), further transformation of H_2_O_2_ might be taken over by other components of the network.

## 5. Conclusion

Here, the alterations in physiological parameters and PSII proteins of *T. pseudonana* cultured at elevated temperatures in response to UVR were comprehensively studied. It was found that the rise of temperature reduced PSII inhibition resulting from UVR mainly by facilitating the PSII repair cycle, in which we found that high temperature maintained higher PsbD pool, and worked synergistically with UVR provoking fast removal of PsbA protein. Irrespective of radiation treatments, high temperature stimulated the induction of SOD activity but induced less NPQs, indicating that temperature promoted repair and protection to a greater extent than inducing damage, and, thus, there was less net UVR damage at higher temperatures. However, it is not known whether the effects resulting from interactions between UVR and ocean warming would be unique to *T. pseudonana* or are characteristic of other phytoplankton taxa as well. Furthermore, decreased nutrient availability as a consequence of enhanced stratification by the increasing ocean surface temperature, will also affect the sensitivity of phytoplankton to UVR (Aranguren-Gassis et al., 2019). The interactions between these factors are almost certainly complex, and therefore figuring out how algae respond requires substantial future studies of these interactions.

## Data availability statement

The original contributions presented in the study are included in the article/supplementary material, further inquiries can be directed to the corresponding author.

## Author contributions

ZX: Conceptualization, Funding acquisition, Investigation, Writing – original draft. SY: Conceptualization, Data curation, Formal analysis, Methodology, Software, Writing – review & editing. ML: Formal analysis, Writing – review & editing. MB: Software, Writing – review & editing. HW: Funding acquisition, Project administration, Writing – review & editing.
